# Distinct 5′ UTR Requirements for Translation of the Bicistronic X/P mRNA Among Avian Orthobornaviruses

**DOI:** 10.1111/1348-0421.70031

**Published:** 2025-12-07

**Authors:** Meng‐Chi Wu, Takehiro Kanda, Ryo Komorizono, Madoka Sakai, Alexander Leacy, Leonardo Susta, Akiko Makino, Keizo Tomonaga

**Affiliations:** ^1^ Laboratory of RNA Viruses, Department of Virus Research, Institute for Life and Medical Sciences Kyoto University Kyoto Japan; ^2^ Department of Mammalian Regulatory Network, Graduate School of Biostudies Kyoto University Kyoto Japan; ^3^ Department of Molecular Virology, Graduate School of Medicine Kyoto University Kyoto Japan; ^4^ Department of Microbiology and Infection, Infection and Advanced Research Center (UTOPIA) The University of Tokyo Pandemic Preparedness, The University of Tokyo Tokyo Japan; ^5^ Department of Pathobiology, Ontario Veterinary College University of Guelph Guelph Canada

**Keywords:** 5′ UTR, avian bornavirus, bicistronic mRNA, gene expression, parrot bornavirus

## Abstract

Orthobornaviruses express X and the phosphoprotein (P) from a bicistronic X/P mRNA, and these proteins regulate polymerase activity. In mammalian orthobornaviruses, the 5′ untranslated region (5′ UTR) of the X/P mRNA controls the translational balance between X and P and thereby promotes efficient replication. Avian bornaviruses (ABVs) belong to two clades, clade‐2 and ‐3, that differ in the structure and length of the 5′ UTR of the X/P mRNA. However, the functional consequences of these differences remain unclear. Using reverse genetics, we generated chimeric viruses by reciprocally exchanging the 5′ UTR of the X/P mRNA among clade 2 parrot bornavirus 5 (PaBV‐5) and aquatic bird bornavirus 1 (ABBV‐1) and clade 3 PaBV‐4. In PaBV‐5, a long 5′ UTR with a stem–loop and an upstream ORF was required to maintain the X‐to‐P translational balance. On the other hand, replacing the 5′ UTR of the X/P mRNA from PaBV‐5 with that from PaBV‐4 reduced X expression and markedly impaired viral growth. However, PaBV‐4 tolerated the 5′ UTR of the X/P mRNA from PaBV‐5 without detectable effects on translation or replication, which suggests that translation of PaBV‐4 X/P mRNA does not depend on the origin of the 5′ UTR. Furthermore, ABBV‐1 replicated efficiently with the 5′ UTR of the X/P mRNA from PaBV‐5 but was strongly attenuated with that from PaBV‐4. Taken together, these results demonstrate a clade‐dependent requirement for the 5′ UTR for translation of the X/P mRNA and provide novel insights into the evolution of translational control in orthobornaviruses.

**Abbreviations**
ABBV‐1: aquatic bird bornavirus 1
ABV: avian bornavirus
BoDV‐1: Borna disease virus 1
ORF: open reading frame
P: phosphoprotein
PaBV‐4: parrot bornavirus 4
PaBV‐5: parrot bornavirus 5
uORF: upstream open reading frame
UTR: untranslated region
vRNP: viral ribonucleoprotein complex
WT: wild type
X: accessory protein

## 1 Introduction

Orthobornaviruses are nonsegmented, negative‐sense, single‐stranded RNA viruses in the genus *Orthobornavirus* within the family *Bornaviridae* [1, 2]. The genus comprises Borna disease virus 1 (BoDV‐1) and variegated squirrel bornavirus 1 (VSBV‐1) from mammals, which cause fatal encephalitis in humans [3, 4, 5], together with avian bornaviruses (ABVs) that infect a wide range of birds, including members of the *Psittaciformes* [6]. Since the discovery of ABVs in 2008 [7, 8], at least 15 ABVs spanning five species have been identified [1, 2, 9]. These include parrot bornavirus 2 and 4 (PaBV‐2 and PaBV‐4), which are major etiological agents of proventricular dilatation disease (PDD), a fatal neurological and gastrointestinal disorder that represents a global infectious threat to psittacine birds [10, 11, 12, 13, 14].

**Figure 1:**
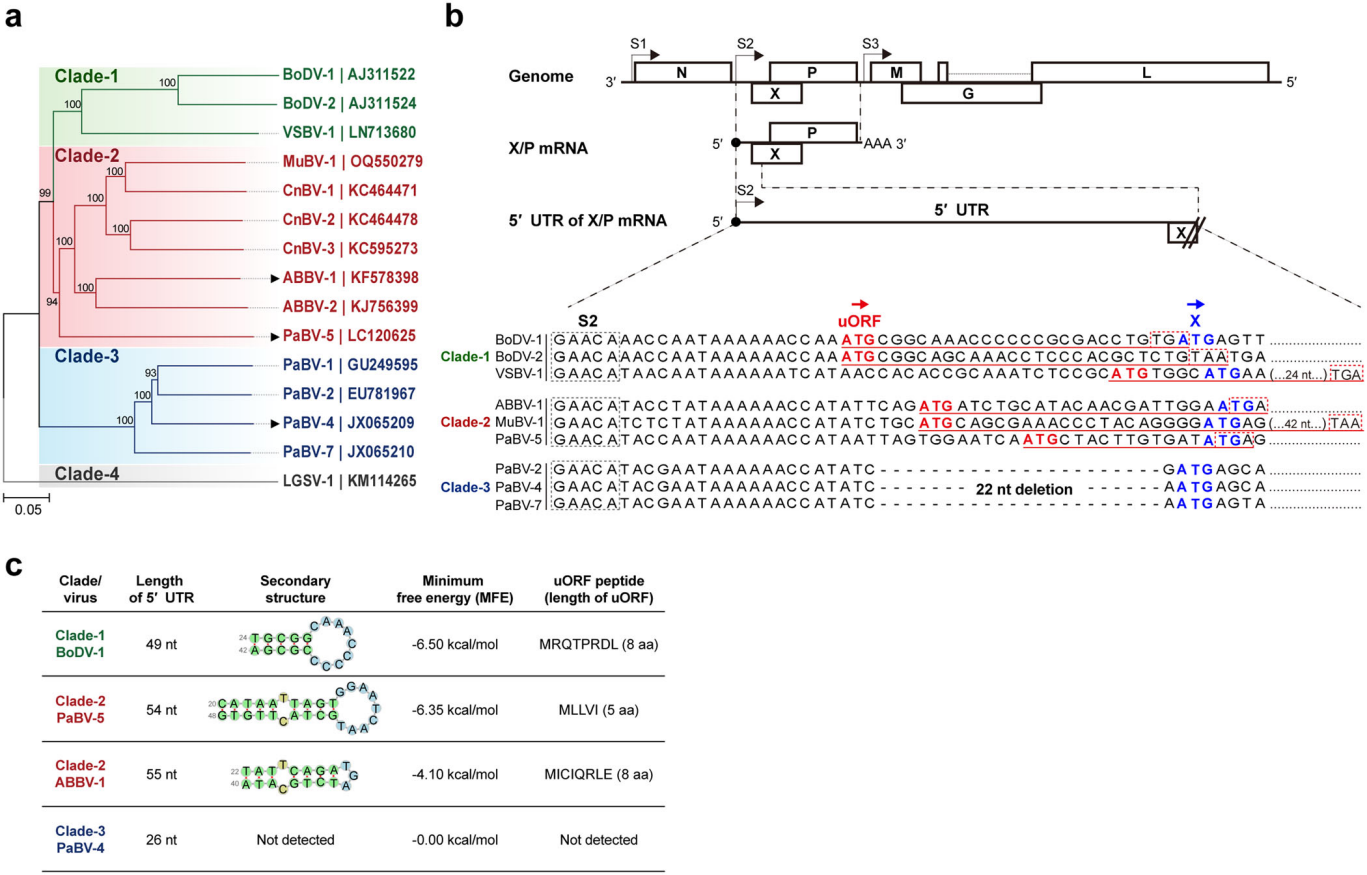
5′ UTR features of the X/P bicistronic mRNA across orthobornaviruses. (a) Phylogenetic tree of *orthobornaviruses* based on genomic sequences excluding the 5′ and 3′ terminal regions. Representative viruses from each clade are shown. Accession numbers are indicated on the right. The tree was constructed by the neighbor‐joining method with the Jukes‐Cantor model and 10,000 bootstrap replicates in MEGA12. The viruses characterized in this study are marked by arrowheads. (b) Schematic of the genome organization and the location of the 5′ UTR of the X/P bicistronic mRNA. Transcriptional start signals (S1−S3) are indicated. An alignment of the 5′ UTRs from representative viruses from each clade is shown, with S2 boxed by black dashed lines. For the putative uORFs, start codons are shown in bold red, coding regions are underlined in red, and stop codons are boxed with red dashed lines. The start codons of the X ORF are shown in bold blue. (c) Summary of the 5′ UTR properties for BoDV‐1 (clade‐1), PaBV‐5 and ABBV‐1 (clade‐2), and PaBV‐4 (clade‐3): length of 5′ UTR, predicted secondary structures, minimum free energy (MFE; kcal/mol), and putative uORF peptides (single‐letter amino acid abbreviations and peptide length) are listed. For PaBV‐4, no secondary structure or uORF was detected. ABBV, aquatic bird bornavirus; BoDV, Borna disease virus; CnBV, canary bornavirus; LGSV‐1, Loveridge's garter snake virus 1; MuBV‐1, munia bornavirus 1; PaBV, parrot bornavirus; VSBV‐1, variegated squirrel bornavirus 1.

Orthobornaviruses can be divided into four clades based on phylogeny inferred from genome sequences [15, 16]. ABVs occupy two of these, clade‐2 and clade‐3, each forming several clusters of closely related viruses that comprise distinct species (Figure 1a). Clade‐2 includes ABVs from non‐psittacine hosts such as aquatic bird bornaviruses (ABBVs) and canary bornaviruses (CnBVs) and also includes PaBV‐5, which has been reported only from limited regions in Asia, Turkey, and the United States [15, 16, 17, 18, 19, 20]. Clade‐3 comprises the majority of PaBVs, such as PaBV‐2 and PaBV‐4, which are the main causes of PDD [14, 21, 22].

The genomes of orthobornaviruses uniformly encode six proteins: nucleoprotein (N), accessory protein (X), phosphoprotein (P), matrix protein (M), glycoprotein (G), and large polymerase (L), in the order from the 3′ to 5′ end [23]. In contrast, viruses in different clades display characteristic sequence features [16]. For example, sequences corresponding to the 5′ untranslated region (5′ UTR) of the bicistronic X/P mRNA, which encodes both X and P proteins, are highly informative clade markers [20, 24, 25]. Viruses in clade‐1 and clade‐2 have long 5′ UTRs of 50 nucleotides (nt) or more that contain a predicted short‐stem loop and an upstream ORF (uORF) [26]. In clade‐3, the 5′ UTR is short at 30 nt or less and lacks both the stem‐loop and the uORF [24, 25] (Figure 1b,c). Our previous study showed that the 5′ UTR of the X/P mRNA of BoDV‐1 binds the host RNA helicase DEAD‐box RNA helicase 21 (DDX21) and mediates translational control of X and P proteins through the stem‐loop and the uORF, which supports efficient viral replication in infected cells [26]. The X is a negative regulator of orthobornavirus polymerase activity, and the P functions as an essential cofactor of the viral polymerase complex [27, 28, 29, 30]. Control of the translational balance between X and P by the 5′ UTR of the X/P mRNA is, therefore, considered crucial for the viral replication cycle [27, 28, 29, 30, 31]. However, as seen in clade‐3 ABVs, some viruses lack an apparent functional structure in the 5′ UTR, so the role of the 5′ UTR of the X/P mRNA in ABVs remains unresolved. Because clade‐2 and clade‐3 ABVs differ in 5′ UTR sequences [24, 25] (Figure 1b,c), and these clades appear to differ in global spread and in the frequency of pathogenic outcomes, it is important to determine how the regulation of the 5′ UTR influences replication and transmission in ABVs with distinct pathogenic properties.

In this study, we analyzed how differences in the 5′ UTR of X/P mRNA affect replication of clade‐2 and clade‐3 ABVs. In addition to recombinant PaBV‐4 (rPaBV‐4) we previously reported [32], in this study, we established reverse genetics systems to recover recombinant PaBV‐5 and ABBV‐1 (rPaBV‐5 and rABBV‐1) from clade‐2 and generated chimeric viruses, in which the 5′ UTRs of the X/P mRNAs were reciprocally exchanged. The analyses showed that in PaBV‐5, the long 5′ UTR that contains the stem‐loop and the uORF is essential for translational control of X and P proteins in avian cells. In contrast, efficient translational control in avian cells did not strictly require the long 5′ UTR in PaBV‐4. Studies with another clade‐2 virus, ABBV‐1, which infects waterfowl, also supported a role for a long 5′ UTR of the X/P mRNA in its efficient replication.

These findings suggest that clade‐2 ABVs depend on translational control embedded in the 5′ UTR of the X/P mRNA to maintain the balance between X and P expression, whereas clade‐3 ABVs, including a pathogenic PaBV‐4, have evolved a distinct mechanism that supports efficient replication in avian cells even in the absence of putative regulatory sequences in the 5′ UTR of the X/P mRNA. Together with the reverse genetics systems for clade‐2 ABVs established here, this study contributes to revealing the basic virology of ABVs and will support future studies toward the development of antivirals and infection prevention strategies.

## 2 Materials and Methods

### 2.1 Cell Culture

QT6 (quail fibroblast cells, ATCC CRL‐1708), QM7 (quail muscle clone 7, quail fibroblast cells, ATCC CRL‐1962), and Human embryonic kidney (HEK) 293 T (ATCC CRL‐3216) cells were cultured in Dulbecco's modified Eagle's medium (DMEM) (Thermo Fisher Scientific, USA) with 10% heat‐inactivated fetal bovine serum (FBS) (Thermo Fisher Scientific, USA) and 1% penicillin‐streptomycin‐amphotericin B solution (Fujifilm, Japan). QM7 cells stably expressing the SV40 large T antigen (QM7‐T) were generated by using the PiggyBac Transposon Vector System (System Biosciences, USA), as previously described [32]. Blasticidin‐resistant QT6 cells were generated by lentiviral transduction of a vector encoding the blasticidin‐resistance gene, as previously described [32]. Cells were maintained in a humidified atmosphere containing 5% CO_2_ at 37°C.

### 2.2 Plasmid Construction and Transfection

Viral sequences were obtained from the GenBank database: PaBV‐4 (accession no. JX065209), PaBV‐5 (accession no. LC120625), and ABBV‐1 (accession no. KF578398). cDNA clones corresponding to the X/P mRNA for PaBV‐4 and PaBV‐5 were constructed into the pcDNA3 vector as previously described [26]. The extraneous sequence from the plasmid between the transcription start site and the viral 5′ UTR of X/P mRNA was subsequently removed. Cultures of QM7‐T grown overnight were transfected with 0.6 μg of plasmid to express X and P, using Lipofectamine 3000 (Invitrogen, USA). Cells were cultured for 18 h before western blot analysis. To prepare the plasmids for the reverse genetics system, full‐length cDNAs of viral antigenomic RNA for clade‐2 (PaBV‐5 and ABBV‐1) and clade‐3 (PaBV‐4) were cloned into pCAG‐HRSV3, each flanked by a hammerhead ribozyme (HamRz) at the 5′ end and a hepatitis delta virus ribozyme (HdvRz) at the 3′ end [33, 34]. Besides, five helper plasmids (pCAGGS‐N, P, L, M, and G) for each virus and BoDV‐1 (accession no. AJ311522) were cloned into the pCAGGS vector [32, 34]. Mutant versions of these plasmids were created using PCR‐based site‐directed mutagenesis.

### 2.3 Reverse Genetics for ABVs and Cell‐Free Virus Preparation

The reverse genetics procedure was modified from our previous report [32, 34]. Briefly, 293 T cells were seeded in six‐well plates and transfected with 2 µg of full‐length viral cDNA plasmid and five helper plasmids encoding N, P, L, M, and G (0.5 µg pCAGGS‐N, 0.025 μg pCAGGS‐P, 0.25 μg pCAGGS‐L, 0.03 μg pCAGGS‐M, and 0.01 μg pCAGGS‐G) using the TransIT‐293 Dynamic Delivery System (Mirus Bio, USA). At 3 days post‐transfection (dpt), the transfected cells were cocultured with blasticidin‐resistant QT6 cells and passaged every 3 or 4 days. Transfected 293 T cells were subsequently eliminated by treating the culture medium with 8.0 μg/mL blasticidin (InvivoGen, USA) for 1 week. For cell‐free virus preparation, cocultured cells were collected by trypsinization, centrifuged, and resuspended in OptiMEM (Invitrogen, USA). The cells were then sonicated using a Bioruptor II (Sonic Bio, Japan), followed by centrifugation at 1200×*g* for 25 min at 4°C. The resulting supernatant was collected and stored at −80°C as the viral stock.

### 2.4 Immunofluorescence Assay (IFA)

Cells after the removal of the culture medium were fixed with 4% paraformaldehyde (Nacalai Tesque, Japan) for 15 min, then permeabilized in phosphate‐buffered saline (PBS) containing 0.5% Triton X‐100 (Wako, Japan) and 5% bovine serum albumin (Sigma‐Aldrich, USA) for another 15 min. The cells were incubated with anti‐BoDV‐1 N (rabbit polyclonal HB01) at a 1:4000 dilution or anti‐BoDV‐1 P antibody (rabbit polyclonal 25 F/10) at a 1:3000 dilution, then with 1:1000 diluted Alexa Fluor 555 or 488‐conjugated anti‐rabbit IgG antibody (Thermo Fisher Scientific, USA) and 300 nM 4′, 6‐diamidino‐2‐phenylindole (DAPI) (Merck, Germany). Fluorescence images were acquired with an ECLIPSE TE2000‐U fluorescence microscope (Nikon, Japan) and an ECLIPSE Ti confocal laser scanning microscope (Nikon, Japan).

### 2.5 Growth Kinetics Assay

Infected QT6 cells were cocultured with uninfected QT6 cells at a ratio of 5:95, and passaged every 3 or 4 days [32]. The ratios of infected cells were determined by IFA.

### 2.6 Titration Assay

Cell‐free virus was titrated on QT6 cells in a 96‐well plate format using IFA as described previously [35]. Ten‐fold serial dilutions of virus suspension in OptiMEM (Invitrogen, USA) were added to each well (50 μL/well) and incubated at 37°C for 1 h. After 3 days of incubation, IFA was performed to detect infected cells, and viral titers were calculated according to the published method [36].

### 2.7 Reverse Transcription Quantitative PCR (RT‐qPCR)

Total RNA was extracted from cultured cells using the NucleoSpin RNA Plus kit (Macherey‐Nagel, Germany) following the manufacturer's protocol. For reverse transcription (RT), 1 μg of total RNA was converted into cDNA in a 20 μL reaction volume using the Verso cDNA Synthesis Kit (Thermo Fisher Scientific, USA). RT was performed using oligo‐dT primers to detect viral mRNA or viral genome‐specific primers to detect viral genomic RNA. For quantitative PCR (qPCR) analysis, reactions were performed using Luna Universal qPCR Master Mix (New England Biolabs, USA) on a CFX Connect real‐time system (Bio‐Rad, USA). The primers used in this study are listed in Table 1. The relative expression levels of viral RNA were calculated via the relative quantification method, with glyceraldehyde‐3‐phosphate dehydrogenase (GAPDH) mRNA serving as the internal reference.

**Table 1:** Sequences of primers used for RT‐qPCR.

Target gene	Forward primer (5′ to 3′)	Reverse primer (5′ to 3′)
PaBV‐4‐N	CACGATTTAGACGGCGAGAA	GCGAATCCGGTTACACCTATTA
PaBV‐4‐P^a^	CTAACTGTGCCCGTCGAGAA	TTAAGCCCCTCTGCCTCGAT
PaBV‐4‐gRNA‐specific RT^a^	GTTGCGGTAACAACCAACCAGCAAC	—
PaBV‐5‐N	CCGCCTGGAAAGGAGTTTAT	AACTGCAGGGATGGTTATGG
PaBV‐5‐P	GCACTGACTCAACCTGTGGA	AGTCCCTCATTCTCAATCAT
PaBV‐5‐gRNA‐specific RT	GTTGCGTTAACAACAAACCAGCGACC	—
ABBV‐1‐N	GTTACGCGCAGATGACTACTT	GTACGCGACAGCTGGAATAA
ABBV‐1‐P	GCTCTGACACAACCAGTCGAAC	AAACCTTCAGCTTCGACCATGC
ABBV‐1‐gRNA‐specific RT	GTTGCGGTAACAACAAACCAACCTCC	—
Quail GAPDH^a^	GCAACCGTGTTGTGGACTTG	GGGAACAGAACTGGCCTCTC

Abbreviations: ABBV‐1, aquatic bird bornavirus 1; GAPDH, glyceraldehyde‐3‐phosphate dehydrogenase; gRNA, genomic RNA; N, nucleoprotein; P, phosphoprotein; PaBV‐4, parrot bornavirus 4; PaBV‐5, parrot bornavirus 5; RT, reverse transcription.

^a^ Primer sequences from Komorizono et al. [32].

### 2.8 Western Blot Analysis

QM7‐T cells transfected with expression plasmids or infected cells were lysed using a 2x sample buffer (Nacalai Tesque, Japan) and boiled at 95°C for 10 min. Cell lysates were separated by SDS‐PAGE on an ePAGEL (ATTO Corporation, Japan) and then transferred to Trans‐Blot Turbo Mini nitrocellulose membranes (Bio‐Rad, USA). The membranes were blocked in Tris‐buffered saline (TBS) containing 0.1% Tween‐20 with 5% (w/v) skim milk and then probed with primary antibodies: anti‐BoDV‐1 N (rabbit polyclonal HB01) at a 1:1000 dilution, anti‐BoDV‐1 P (rabbit polyclonal HB03) at a 1:1000 dilution, anti‐BoDV‐1 X (rabbit polyclonal H1766) at a 1:100 dilution, or anti‐alpha‐tubulin (Sigma‐Aldrich, USA) at a 1:10,000 dilution. Following incubation, the membranes were incubated with horseradish peroxidase (HRP)‐conjugated anti‐mouse IgG or anti‐rabbit IgG secondary antibodies (Jackson ImmunoResearch, USA) at a 1:10,000 dilution. Protein bands were detected using Clarity Western enhanced chemiluminescence (ECL) substrate (Bio‐Rad, USA), and chemiluminescence signals were visualized with a Fusion Solo S imaging system (Vilber, France).

### 2.9 RNA Structure Prediction

The nucleotide sequence of the full‐length 5′ UTR of X/P mRNA was analyzed using the minimum free energy (MFE) method with RNAfold (ViennaRNA Package 2.0).

### 2.10 Statistical Analysis

Analyses were conducted using GraphPad Prism software (version 10), with statistical tests indicated in the figure legends.

## 3 Results

### 3.1 The 5′ UTR of the X/P mRNA Regulates Translation of PaBV‐5 X But Has Little Effect on PaBV‐4 X

**Figure 2:**
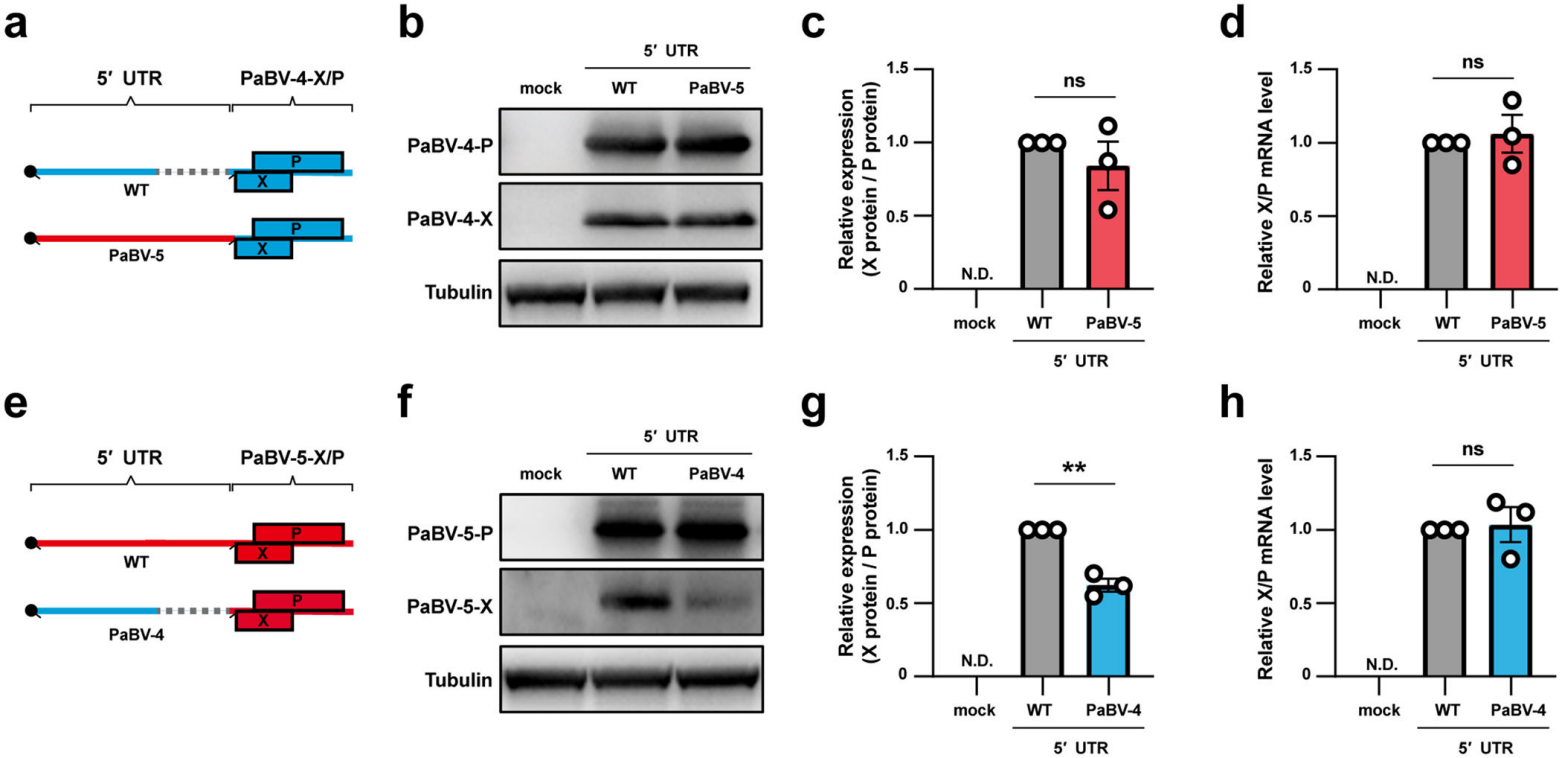
5′ UTR‐dependent control of X expression in plasmid‐based assays for PaBV‐4 and PaBV‐5. (a) Schematics of PaBV‐4 X/P mRNA harboring either the wild‐type (WT) or PaBV‐5‐derived 5′ UTR. (b−d) QM7‐T cells were transfected with 0.6 μg of each PaBV‐4 X/P plasmid. (b) X and P protein expression at 18 h post‐transfection (hpt) detected by western blot analysis. (c) The X‐to‐P protein ratio was quantified from band intensities using ImageJ. (d) Total RNA extracted at 18 hpt; X/P mRNA levels measured by RT‐qPCR. (e) Schematic diagrams of the PaBV‐5 X/P mRNA harboring either the WT or PaBV‐4‐derived 5′ UTR. (f−h) As in (b–d) for PaBV‐5: (f) immunoblot at 18 hpt; (g) X‐to‐P protein ratio quantification; (h) X/P mRNA levels by RT‐qPCR at 18 hpt. Data represent the means ± standard errors of the means (±SEM) from three independent experiments. (c, d, g, h) Statistical significance was determined by an unpaired *t*‐test; ns, not significant; **p < 0.01.

To examine how the 5′ UTR of the X/P mRNA affects the translation of X and P proteins in PaBV‐4 and PaBV‐5, we constructed X/P expression plasmids for each virus in which the coding region was placed under either the cognate or the heterologous 5′ UTR from the other virus (Figure 2a,e). Upon transfection into QM7‐T cells, PaBV‐4 constructs bearing either 5′ UTR produced similar amounts of X and P proteins and yielded comparable X‐to‐P ratios (Figure 2b,c). RT‐qPCR confirmed equivalent X/P transcript levels in both plasmids (Figure 2d). In contrast, PaBV‐5 constructs carrying the PaBV‐4 5′ UTR showed reduced production of X compared with the native PaBV‐5 5′ UTR (Figure 2f), resulting in an approximately twofold decrease in the X‐to‐P ratio (Figure 2g). RT‐qPCR showed comparable X/P transcript levels (Figure 2h), indicating that the difference arose at the translational level rather than from changes in transcript abundance. These results indicated that, in a plasmid‐based system, translation of PaBV‐4 X and P proteins appeared largely insensitive to 5′ UTR origin, whereas that of PaBV‐5 X depends on features within its native 5′ UTR.

### 3.2 Reciprocal Exchange of the 5′ UTR of the X/P mRNA Impairs Replication of PaBV‐5 But Not PaBV‐4

**Figure 3:**
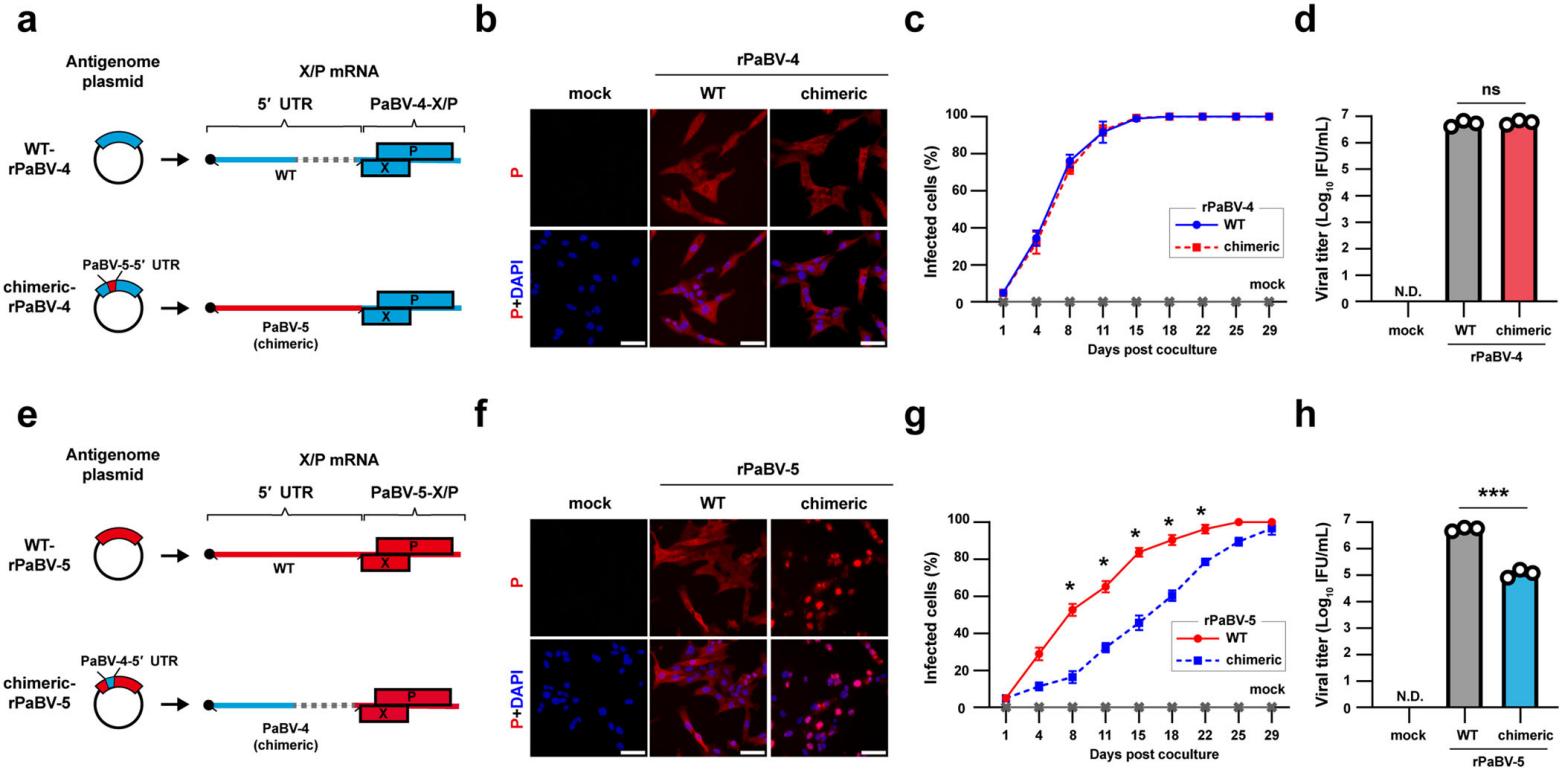
Effects of reciprocal exchange of the 5′ UTR on replication and viral titers of rPaBV‐4 and rPaBV‐5. (a) Schematic diagrams of antigenome plasmids for rPaBV‐4 and carrying either the native (WT) or the PaBV‐5‐derived (chimeric) 5′ UTRs of X/P mRNA. The corresponding X/P mRNAs with the indicated 5′ UTRs are shown on the right. (b) Rescue of WT‐rPaBV‐4 and chimeric‐rPaBV‐4. Infection in QT6 cells was confirmed by IFA. Representative images at 36 days post‐coculture (dpc) are shown. P protein was detected with anti‐BoDV‐1 P antibody (red), and nuclei were counterstained with DAPI (blue). Scale bar, 50 μm. (c) Growth kinetics of rPaBV‐4s. Uninfected and rPaBV‐4‐infected QT6 cells were cocultured and passaged every 3 or 4 days. The percentage of infected cells was measured by IFA. (d) Infectious titers of rPaBV‐4. Viruses rescued at 29 dpc were titrated on QT6 cells. (e) Schematic diagrams of antigenome plasmids for rPaBV‐5 and carrying either the native (WT) or the PaBV‐4‐derived (chimeric) 5′ UTRs of the X/P mRNA. The corresponding X/P mRNAs with the indicated 5′ UTRs are shown on the right. (f−h) As in (b–d) for PaBV‐5 (f) IFA image at 36 dpc. Scale bar, 50 μm. (g) Growth kinetics of WT‐ and chimeric‐rPaBV‐5. (h) Infectious titers of rPaBV‐5. rPaBV‐5s were rescued at 29 dpc. Data represent the means ± SEM from three independent experiments. Statistical significance was determined by two‐way ANOVA with Tukey's multiple‐comparison test (c, g), and unpaired *t*‐test (d, h); ns, not significant; *p < 0.05; ***p < 0.001.

To assess the contribution of the 5′ UTR of X/P mRNA to viral replication, we recovered rPaBV‐4 and rPaBV‐5 in which the X/P mRNA carried either the native 5′ UTR or the heterologous 5′ UTR from the other virus by reverse genetics (Figure 3a,e). For PaBV‐4, both the native construct WT‐rPaBV‐4 and the chimeric construct bearing the PaBV‐5 5′ UTR were rescued (Figure 3b) and showed indistinguishable growth kinetics in QT6 cells (Figure 3c). Virus titers recovered from infected cells were nearly identical for the two rPaBV‐4s (Figure 3d). rPaBV‐5 was rescued using the same protocol as for PaBV‐4 (Figure S1) and recovered WT‐rPaBV‐5 and a chimeric rPaBV‐5 carrying the PaBV‐4 5′ UTR on the X/P mRNA (Figure 3e,f). In QT6 cells, the growth ability of chimeric‐rPaBV‐5 was severely attenuated compared to that of WT‐rPaBV‐5 (Figure 3g). The viral titers recovered at 29 days post‐coculture (dpc) were approximately hundredfold lower than those of WT‐rPaBV‐5 (Figure 3h). These results indicated that efficient replication of PaBV‐5 depends on the native 5′ UTR context of the X/P mRNA, whereas replication of PaBV‐4 was not appreciably altered by 5′ UTR origin.

## 4 The 5′ UTR of X/P mRNA Is Required to Maintain the Translational Balance Between X and P Proteins in PaBV‐5

**Figure 4:**
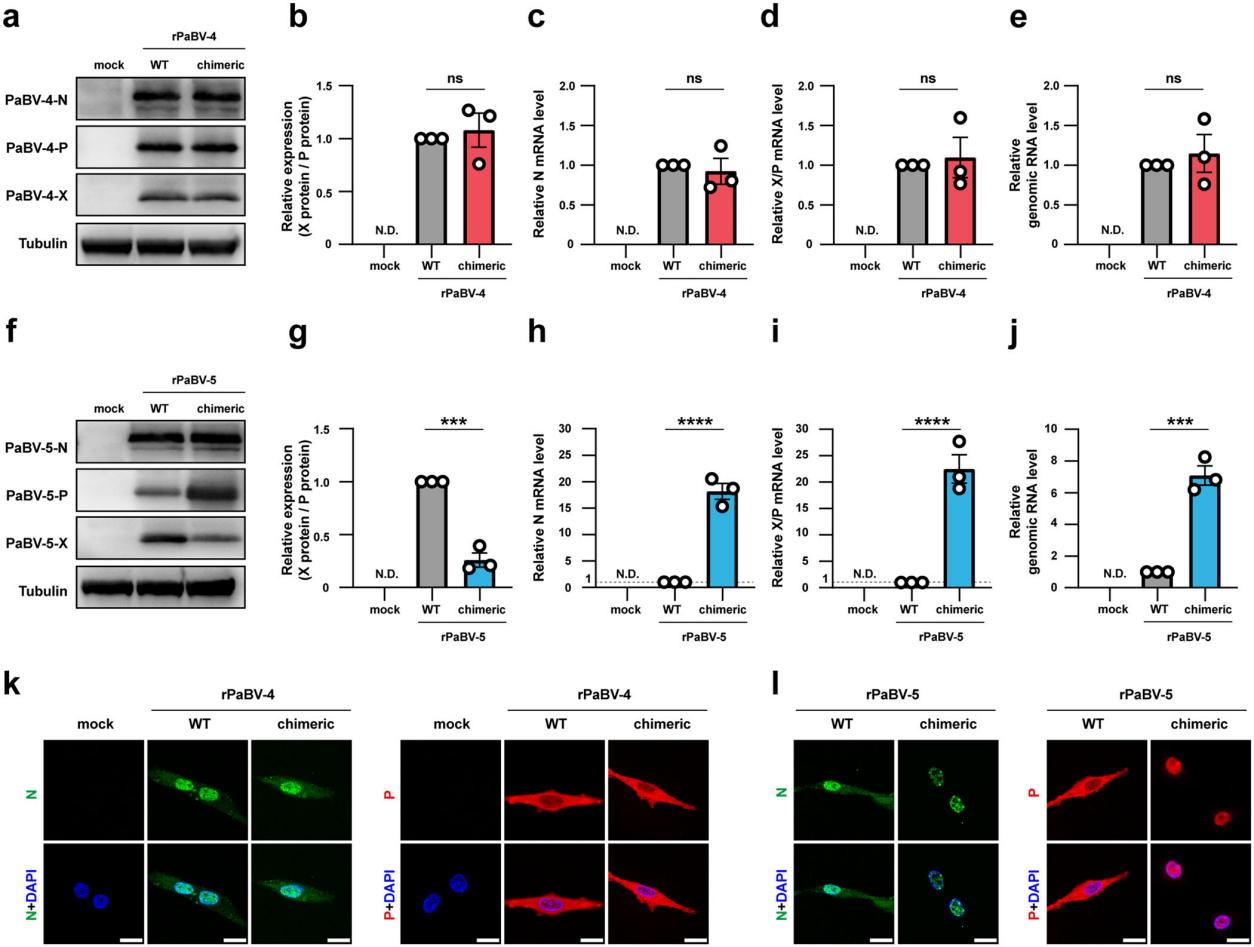
Effects of the 5′ UTR of X/P mRNA on viral gene expression and protein localization in rPaBV‐4 and rPaBV‐5. (a and b) Expression of rPaBV‐4 N, X, and P proteins in QT6 cells. (a) Viral proteins were detected by western blot analysis. (b) X‐to‐P protein ratios quantified from band intensities using ImageJ. (c−e) Relative RNA levels in rPaBV‐4‐infected QT6 cells: (c) N mRNA, (d) X/P mRNA, and (e) genomic RNA. Viral RNAs were quantified by RT‐qPCR. (f and g) Expression of rPaBV‐5 N, X, and P proteins in QT6 cells. (f) Western blot analysis. (g) X‐to‐P protein quantified as in (b). (h−j) Relative RNA levels in rPaBV‐5‐infected QT6 cells: (h) N mRNA, (i) X/P mRNA, and (j) genomic RNA, measured by RT‐qPCR. (k and l) Subcellular localization of N (left) and P (right) in QT6 cells. Cells infected with rPaBV‐4s (k) or rPaBV‐5s (l) were analyzed by IFA and imaged by confocal microscopy. Primary antibodies used are indicated on the left of each panel; nuclei were counterstained with DAPI (blue). Scale bar, 20 μm. Data represent the means ± SEM of three independent experiments. Statistical significance was determined by an unpaired *t*‐test (b−e, g−j); ns, not significant; ***p < 0.001; ****p < 0.0001.

To investigate how the 5′ UTR of the X/P mRNA influences viral gene expression, we compared expression levels of viral proteins and the relative levels of viral RNAs in QT6 cells infected with WT and chimeric viruses. WT‐rPaBV‐4 and chimeric‐rPaBV‐4 produced comparable levels of N, X, and P proteins (Figure 4a), with no significant differences in the X‐to‐P ratio (Figure 4b). The levels of N and X/P mRNAs and genomic RNA were almost identical between WT‐ and chimeric‐rPaBV‐4 (Figure 4c–e). In contrast, the expression level of P increased while that of X decreased in chimeric‐rPaBV‐5‐infected cells compared to WT‐rPaBV‐5‐infected cells (Figure 4f), resulting in a significant reduction in the X‐to‐P ratio (Figure 4g). Although the expression level of N protein was almost identical (Figure 4f), the levels of N mRNA increased by around 18.7‐fold in chimeric‐rPaBV‐5‐infected cells compared to WT‐rPaBV‐5‐infected cells (Figure 4h). The levels of X/P mRNA (Figure 4i) and genomic RNA (Figure 4j) also increased by about 21.0‐ and 6.8‐fold in chimeric‐rPaBV‐5‐infected cells compared to WT‐rPaBV‐5‐infected cells. These results suggest that the native 5′ UTR on the PaBV‐5 X/P mRNA is required to maintain the translational balance between X and P and also contributes to the regulation of the viral transcription and replication.

Our previous studies on BoDV‐1 showed that the relative expression levels of X and P in infected cells are important for regulating the subcellular localization of viral proteins and thereby enabling efficient viral replication in the nucleus [25, 37]. Therefore, we next examined the localization of viral proteins in WT‐ and chimeric virus‐infected cells. In rPaBV‐4‐infected cells, the N and P proteins localized in both the nucleus and the cytoplasm (Figure 4k). On the other hand, in WT‐rPaBV‐5‐infected cells, N and P proteins also localized to both the nucleus and the cytoplasm, whereas they were predominantly restricted in the nucleus in chimeric‐rPaBV‐5‐infected cells (Figure 4l). These results indicate that the native 5′ UTR of the PaBV‐5 X/P mRNA influences the proper subcellular localization of N and P.

### 4.1 Inter‐Clade But Not Intra‐Clade Exchange of the 5′ UTR of the X/P mRNA Impairs Replication of Clade‐2 Orthobornaviruses

**Figure 5:**
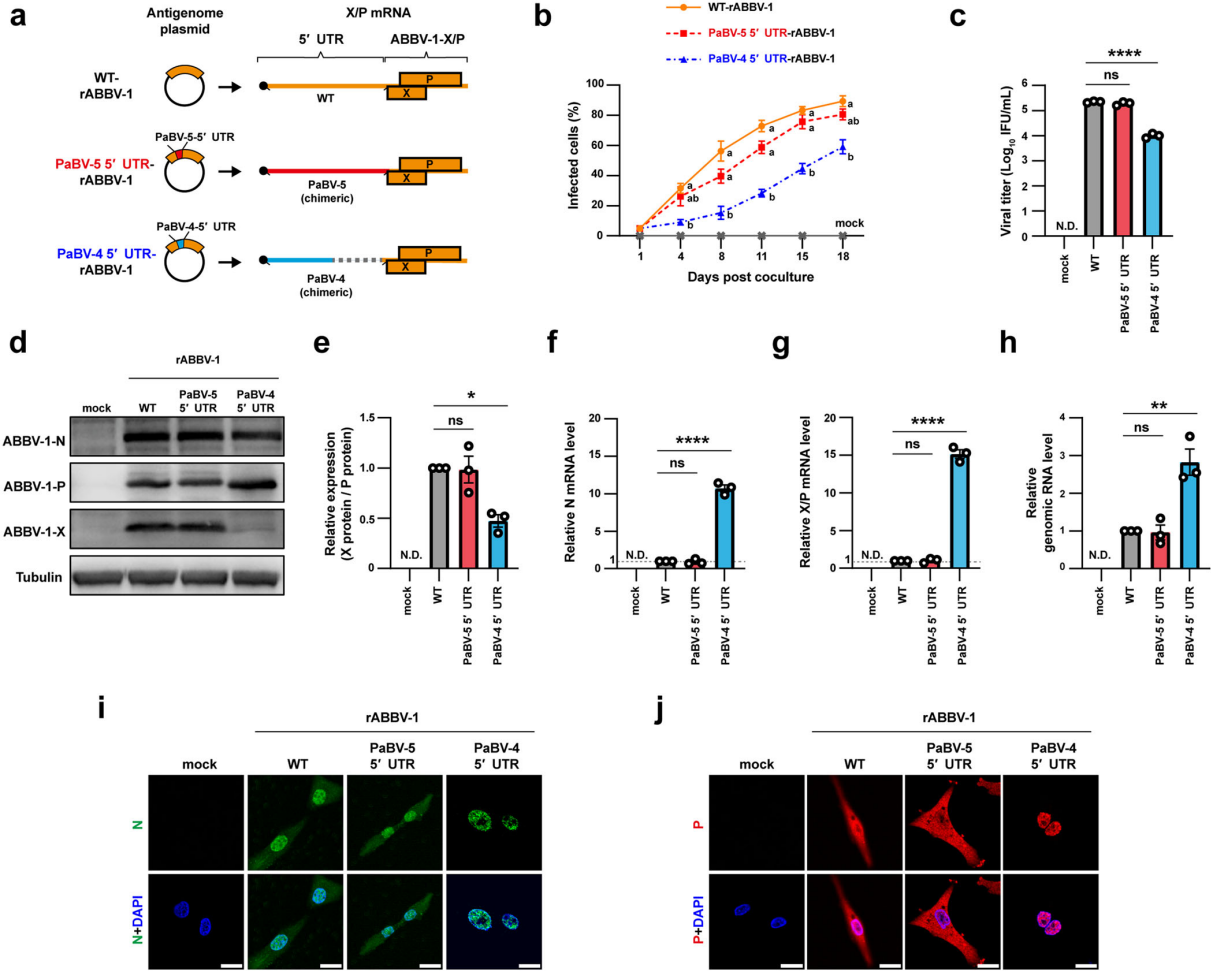
A long 5′ UTR of X/P mRNA is required for replication of the clade‐2 ABBV‐1 via reciprocal exchange with PaBV‐5 and PaBV‐4. (a) Schematic diagrams of antigenome plasmids of rABBV‐1 carrying either the native (WT), the PaBV‐5‐derived (PaBV‐5 5′ UTR), or the PaBV‐4‐derived (PaBV‐4 5′ UTR) 5′ UTR of the X/P mRNA. The corresponding X/P mRNAs with the indicated 5′ UTRs are shown at the right. (b) Growth kinetics of rABBV‐1 in QT6 cells. Uninfected and rABBV‐1‐infected QT6 cells were cocultured and passaged every 3 or 4 days. Infection ratios were measured by IFA. (c) Infectious titers of rABBV‐1. Each virus was harvested when the infection ratio exceeded 50%, and the titers were determined on QT6 cells. (d and e) Expression of rABBV‐1 N, X, and P proteins in QT6 cells. (d) Western blot analysis; (e) X‐to‐P protein ratios quantified from band intensities using ImageJ. (f−h) Relative RNA levels in rABBV‐1‐infected QT6 cells; (f) N mRNA, (g) X/P mRNA, and (h) genomic RNA, measured by RT‐qPCR. (i and j) Subcellular localization of N (i) and P (j) proteins in infected QT6 cells analyzed by IFA; nuclei were counterstained with DAPI (blue). Scale bar, 20 μm. Data represent the means ± SEM of three independent experiments. Statistical significance was determined by two‐way (b) and one‐way ANOVA (c, e−h), and Tukey's multiple‐comparison test (b, c, e−h); ns, not significant; *p < 0.05; **p < 0.01; ****p < 0.0001. In (b), different letters (a, b) next to the bars indicate statistically significant differences at *p* < 0.05.

To test whether the dependence on the native 5′ UTR of the X/P mRNA observed in PaBV‐5 also applies to other clade‐2 viruses, we established a reverse genetics system for ABBV‐1 (Figure S1) and recovered wild‐type rABBV‐1 (WT‐rABBV‐1). We then generated chimeric rABBV‐1 carrying either the PaBV‐5‐derived 5′ UTR (PaBV‐5 5′ UTR‐rABBV‐1) or the PaBV‐4‐derived 5′ UTR (PaBV‐4 5′ UTR‐rABBV‐1) (Figure 5a). Compared with WT‐rABBV‐1, PaBV‐5 5′ UTR‐rABBV‐1 showed similar growth in QT6 cells, whereas PaBV‐4 5′ UTR‐rABBV‐1 was markedly attenuated (Figure 5b). Viral titers recovered from WT‐rABBV‐1‐ and PaBV‐5 5′ UTR‐rABBV‐1‐infected cells were comparable, whereas titers from PaBV‐4 5′ UTR‐rABBV‐1‐infected cells were lower by approximately 100‐fold (Figure 5c).

Compared with WT‐rABBV‐1‐infected cells, expression levels of N, X, and P in PaBV‐5 5′ UTR‐rABBV‐1‐infected cells were comparable, whereas the expression level of P protein increased while that of X protein decreased in PaBV‐4 5′ UTR‐rABBV‐1‐infected cells, resulting in a significant reduction in the X‐to‐P ratio (Figure 5d,e). In addition, the levels of N mRNA, X/P mRNA, and genomic RNA increased by approximately 10.7‐, 15.1‐, and 2.8‐fold in PaBV‐4 5′ UTR‐rABBV‐1‐infected cells compared with WT‐rABBV‐1‐infected cells (Figure 5f–h). N and P proteins localized to both the nucleus and the cytoplasm in WT‐rABBV‐1‐ and PaBV‐5 5′ UTR‐rABBV‐1‐infected cells, whereas they were predominantly accumulated in the nucleus in PaBV‐4 5′ UTR‐rABBV‐1‐infected cells (Figure 5i,j). These results indicate that clade‐2 viruses require a clade‐2‐derived 5′ UTR for efficient viral replication and gene expression and that the intra‐clade exchange preserves replication efficiency even when the stem‐loop and uORF sequences differ (Figure 1c).

## 5 Discussion

In this study, we established reverse genetics systems for PaBV‐5 and ABBV‐1 and examined how the 5′ UTR of the X/P mRNA regulates viral gene expression and replication of clade‐2 and clade‐3 ABVs. In rPaBV‐5 and rABBV‐1, replacement of the native 5′ UTR with that of PaBV‐4 decreased expression of X, while increasing that of P, and attenuated viral growth ability (Figures 3, 4, 5). In contrast, rPaBV‐4 tolerated the PaBV‐5 5′ UTR without loss of translational balance and viral replication (Figures 2 and 4). Therefore, the long 5′ UTR in clade‐2 viruses, which contains a putative stem‐loop structure and a uORF, is important for maintaining the proper translational balance between X and P proteins and for efficient viral replication, identifying the clade‐2‐specific 5′ UTR as a key determinant of viral replication.

Plasmid‐based experiments indicated that the 5′ UTR of the X/P mRNA in PaBV‐5 is a key regulator of X and P translation (Figure 2f,g). Using prototype orthobornavirus, BoDV‐1 (clade‐1), we previously demonstrated that the translational balance between X and P is regulated via host DDX21‐mediated stabilization of stem‐loop structure in the 5′ UTR [26]. In brief, phosphorylated DDX21 stabilizes the stem‐loop structure in the 5′ UTR and inhibits ribosomal recognition of the X start codon, thereby suppressing translation of X while facilitating that of P. As P accumulates, it interferes with DDX21 phosphorylation, leading to dissociation of the complex from the 5′ UTR and relieving the block on X translation, which relies on a uORF termination‐coupled reinitiation mechanism [26]. Considering that clade‐2 viruses harbor both a putative stem‐loop structure and a uORF in the 5′ UTR (Figure 1b,c) and that DDX21 is highly conserved between humans and avians [38], translation of X and P in clade‐2 ABVs may be regulated by a mechanism similar to that of BoDV‐1. Although the uORF peptide sequence is not conserved either within or across clades (Figure 1b,c), our previous study has shown that the start and stop codons of the uORF, rather than the encoded peptide, are critical for regulating X expression in BoDV‐1 [26]. Therefore, in PaBV‐5, substitution of its 5′ UTR with the PaBV‐4‐derived 5′ UTR, which lacks both the uORF and the putative stem‐loop, likely disrupted 5′ UTR‐mediated translational regulation and biased translation toward P. It could be important to investigate whether the clade‐2 stem‐loop structure directly interacts with avian DDX21 and whether mutations at phosphorylation sites in PaBV‐5 P affect the translational balance. Notably, whereas our previous work in BoDV‐1 demonstrated that deleting either the uORF or the stem‐loop enhances X translation in mammalian cells [26], this study showed that disrupting these elements by replacing them with the short PaBV‐4 5′ UTR in PaBV‐5 reduced X expression in avian cells (Figures 2f and 4f). Although the basis for this discrepancy is not yet clear, this observation suggests a fundamental difference between mammalian and avian cells in their sensing of viral mRNAs. Host cells possess mechanisms that sense viral mRNAs with short 5′ UTR [39] or specific secondary structures [40] and selectively suppress their translation. If mammalian and avian cells have distinct mechanisms for detecting 5′ UTR length or structure, this could plausibly explain the differential impact on X translation.

In contrast, replacing the 5′ UTR of the X/P mRNA in PaBV‐4 with the PaBV‐5‐derived 5′ UTR affected neither the translational balance nor replication (Figures 2, 3, 4). This finding suggests that PaBV‐4 may have evolved a mechanism that enables its X/P mRNA to evade host‐sensing pathways, allowing efficient translation of X despite its short 5′ UTR. Interestingly, our attempt to rescue a chimeric rPaBV‐4 carrying the PaBV‐5 X and P genes was unsuccessful (data not shown). This also suggests that the translational regulation of PaBV‐4 X/P mRNA may depend on the overall structure of the X/P mRNA or on functional differences in the X or P proteins themselves [25]. Further detailed investigation into the mechanisms governing X/P mRNA translation in clade‐2 and clade‐3 viruses will likely provide novel insights into how avian cells sense viral mRNAs.

In this study, we also demonstrated that both chimeric‐rPaBV‐5 and PaBV‐4 5′ UTR‐rABBV‐1 produced higher levels of viral mRNA and genomic RNA compared to WT‐rPaBV‐5 and WT‐rABBV‐1, despite their attenuated replication capacity (Figures 3, 4, 5). In BoDV‐1, X functions as a negative regulator of polymerase activity through binding to P [28, 30, 37, 41, 42, 43]. Reduced X expression in the chimeric viruses may therefore enhance genomic RNA replication and increase templates for transcription. However, increased transcript abundance did not lead to proportional increases in viral protein levels in chimeric virus‐infected cells (Figures 4 and 5). A decoupling phenomenon between transcriptional output and translation levels has also been reported in vesicular stomatitis virus and dengue virus [44, 45]. This is likely to reflect the limited pool of ribosomes available for viral protein synthesis under conditions that preserve cellular homeostasis. On the other hand, P levels were elevated in chimeric rPaBV‐5‐infected cells at 29 dpc when compared with those in WT‐rPaBV‐5 (Figure 4f). This suggests that the enhanced expression of P driven by the short PaBV‐4‐derived 5′ UTR may accumulate over time and become more pronounced at later stages.

The dysregulation of the X and P expression balance observed in the chimeric clade‐2 viruses that replace the short PaBV‐4 5′ UTR was also evident in the intracellular localization of the viral proteins. In cells infected with chimeric‐rPaBV‐5 or PaBV‐4 5′ UTR‐rABBV‐1, both N and P were restricted to the nucleus (Figures 4l and 5i,j). In contrast, both viral proteins were observed to localize to the cytoplasm in cells infected with wild‐type rPaBV‐5 or rABBV‐1 (Figures 4l and 5i,j). We previously reported that BoDV‐1 P is exported to the cytoplasm when it is co‐expressed with X [25, 37]. We also demonstrated that PaBV‐5 X possesses the ability to promote the cytoplasmic translocation of P, similar to BoDV‐1 X [25]. Because P serves as a protein hub of viral ribonucleoprotein complex (vRNP) [46, 47, 48, 49, 50, 51], accumulation of P can retain vRNP in the nucleus and delay viral propagation [46]. Therefore, the altered intracellular localization of viral proteins in the chimeric‐rPaBV‐5 and PaBV‐4 5′ UTR‐rABBV‐1 likely reflects insufficient X expression and may contribute to attenuation of viral replication through inefficient vRNP export. Further elucidation of the host‐dependent mechanisms underlying the translation and cytoplasmic translocation of X and P will provide insights into the regulation of persistent infection and replication of orthobornaviruses in the nucleus.

## 6 Conclusion

In this study, our data indicate that the long 5′ UTR of the X/P mRNA is an important determinant of replication in clade‐2 ABVs by fine‐tuning gene expression, whereas clade‐3 ABVs show greater tolerance to sequences in this region. These findings provide insights into the differences in pathogenicity and the evolution of diverse ABVs. In addition, reverse genetics systems developed here provide a powerful platform not only for investigating ABV virology but also for developing and evaluating vaccine candidates and antiviral strategies for orthobornaviruses.

## Author Contributions

Conceptualization, methodology, investigation, and writing: Meng‐Chi Wu. Conceptualization, methodology, writing, supervision, and funding acquisition: Takehiro Kanda. Methodology, resources, and funding acquisition: Madoka Sakai, Akiko Makino. Methodology and resources: Ryo Komorizono, Alexander Leacy, Leonardo Susta. Supervision, writing, and funding acquisition: Keizo Tomonaga. Editing and reviewing: all of the authors.

## Ethics Statement

This study did not involve human participants or vertebrate animals. Only established cell lines were used. Therefore, approval by an ethics committee was not required.

## Conflicts of Interest

The authors declare no conflicts of interest.

## Supporting information


**Supporting Figure S1:** Development of a reverse genetics system for clade‐2 ABV. (a) Schematic representation of the reverse genetics procedure. Full‐length ABV antigenomic cDNA plasmids together with five helper plasmids encoding N, P, L, M, and G (N, P, and L were derived from BoDV‐1; M and G were derived from the corresponding ABV) were transfected into 293T cells. At 3 days post‐transfection (dpt), transfected cells were cocultured with blasticidin‐resistant QT6 cells to amplify viruses. Cocultures were passaged twice weekly, with blasticidin added to selectively eliminate 293T cells. (b) Recovery of wild‐type rPaBV‐5 (WT‐rPaBV‐5). 293T cells were transfected with the PaBV‐5 full‐length cDNA and helper plasmids, or with empty plasmids as a mock control. At 3 dpt, the cells were cocultured with QT6 cells and subsequently passaged every 3 or 4 days. Infection ratios were determined by IFA at the indicated days post coculture (dpc). (c) Recovery of wild‐type rABBV‐1 (WT‐rABBV‐1). 293T cells transfected with the ABBV‐1 full‐length cDNA and helper plasmids, or with empty plasmids as a mock control. At 3 dpt, the cells were cocultured with QT6 cells and subsequently passaged every 3 or 4 days. Infection ratios were determined by IFA at the indicated dpc. Data represent means ± SEM of three independent experiments.

## Data Availability

The data that support the findings of this study are available from the corresponding author upon reasonable request.
