# Supplementary material for: Distinct 5′ UTR Requirements for Translation of the Bicistronic X/P mRNA Among Avian Orthobornaviruses
Source: Microbiol Immunol. 2025 Dec 7;70(2):80–90. doi: 10.1111/1348-0421.70031 (PMC12868937; doi:10.1111/1348-0421.70031)
Supplement: Supplementary file 1 — Supporting Figure S1: Development of a reverse genetics system for clade‐2 ABV. (a) Schematic representation of the reverse genetics procedure. Full‐length ABV antigenomic cDNA plasmids together with five helper plasmids encoding N, P, L, M, and G (N, P, and L were derived from BoDV‐1; M and G were derived from the corresponding ABV) were transfected into 293T cells. At 3 days post‐transfection (dpt), transfected cells were cocultured with blasticidin‐resistant QT6 cells to amplify viruses. Cocultures were passaged twice weekly, with blasticidin added to selectively eliminate 293T cells. (b) Recovery of wild‐type rPaBV‐5 (WT‐rPaBV‐5). 293T cells were transfected with the PaBV‐5 full‐length cDNA and helper plasmids, or with empty plasmids as a mock control. At 3 dpt, the cells were cocultured with QT6 cells and subsequently passaged every 3 or 4 days. Infection ratios were determined by IFA at the indicated days post coculture (dpc). (c) Recovery of wild‐type rABBV‐1 (WT‐rABBV‐1). 293T cells transfected with the ABBV‐1 full‐length cDNA and helper plasmids, or with empty plasmids as a mock control. At 3 dpt, the cells were cocultured with QT6 cells and subsequently passaged every 3 or 4 days. Infection ratios were determined by IFA at the indicated dpc. Data represent means ± SEM of three independent experiments. [file MIM-70-80-s001.docx]

**Supporting Information**

**
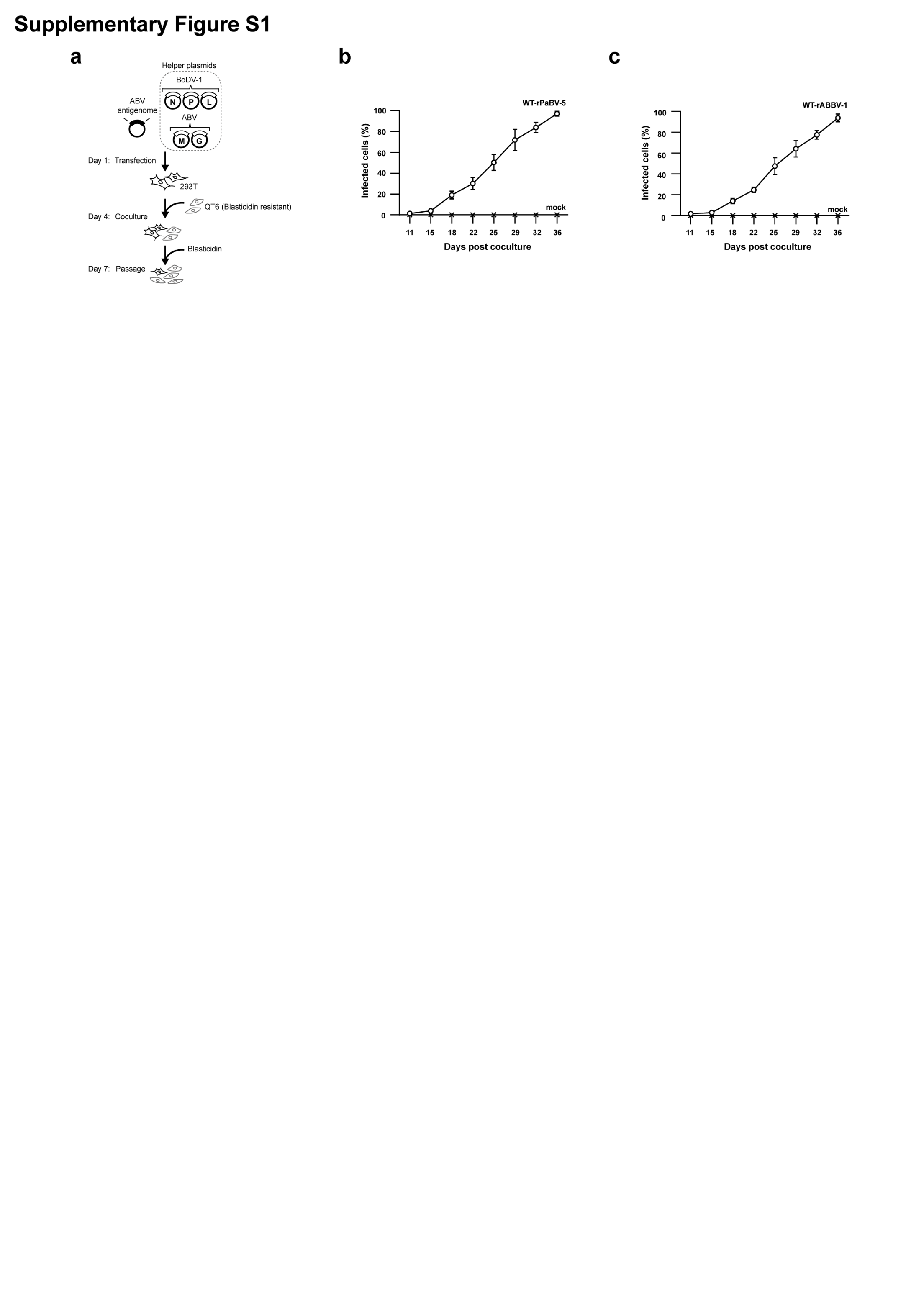
**

**Supplementary Figure S1. Development of a reverse genetics system for clade-2 ABV**

(a) Schematic representation of the reverse genetics procedure. Full-length ABV antigenomic cDNA plasmids together with five helper plasmids encoding N, P, L, M, and G (N, P, and L were derived from BoDV-1; M and G were derived from the corresponding ABV) were transfected into 293T cells. At 3 days post-transfection (dpt), transfected cells were cocultured with blasticidin-resistant QT6 cells to amplify viruses. Cocultures were passaged twice weekly, with blasticidin added to selectively eliminate 293T cells. (b) Recovery of wild-type rPaBV-5 (WT-rPaBV-5). 293T cells were transfected with the PaBV-5 full-length cDNA and helper plasmids, or with empty plasmids as a mock control. At 3 dpt, the cells were cocultured with QT6 cells and subsequently passaged every 3 or 4 days. Infection ratios were determined by IFA at the indicated days post coculture (dpc). (c) Recovery of wild-type rABBV-1 (WT-rABBV-1). 293T cells transfected with the ABBV-1 full-length cDNA and helper plasmids, or with empty plasmids as a mock control. At 3 dpt, the cells were cocultured with QT6 cells and subsequently passaged every 3 or 4 days. Infection ratios were determined by IFA at the indicated dpc. Data represent means ± SEM of three independent experiments.
